# Phase I study of irinotecan and raltitrexed in patients with advanced astrointestinal tract adenocarcinoma

**DOI:** 10.1054/bjoc.2000.1192

**Published:** 2000-06-15

**Authors:** H E R Ford, D Cunningham, P J Ross, S Rao, G W Aherne, T S Benepal, T Price, A Massey, L Vernillet

**Affiliations:** Department of Medicine and Gastrointestinal Unit, Royal Marsden NHS Trust, Downs Road, Sutton, Surrey, SM2 5PT, UK; CRC Centre for Cancer Therapeutics, Institute of Cancer Research, 15 Cotswold Road, Sutton, Surrey, SM2 5NG, UK; Rhône-Poulenc Récherche et Developement, Antony Cedex, France

**Keywords:** irinotecan, raltitrexed, drug combination, phase I, pharmacokinetics

## Abstract

To determine the dose-limiting toxicities (DLT) and maximum tolerated dose (MTD) of irinotecan and raltitrexed given as sequential short infusions every 3 weeks, 33 patients with pretreated gastrointestinal adenocarcinoma (31 colorectal, 2 oesophagogastric) entered this open label dose-escalation study. For the first five dose levels patients received irinotecan 175–350 mg m^–2^followed by raltitrexed 2.6 mg m^–2^. Level VI was irinotecan 350 mg m^–2^plus raltitrexed 3.0 mg m^–2^, level VII was irinotecan 400 mg m^–2^plus raltitrexed 2.6 mg m^–2^; 261 courses were administered. Only one patient at dose levels I–V experienced DLT. At level VI, 5/12 patients experienced DLT: one had grade 3 diarrhoea and lethargy, one had grade 4 diarrhoea and one had lethargy alone. Two others had lethargy caused by disease progression. There was no first-cycle neutropenia. At level VII, 3/6 patients experienced dose-limiting lethargy, one also had grade 3 diarrhoea. Dose intensity fell from over 90% for both drugs at level VI to 83% for irinotecan and 66% for raltitrexed at level VII. Lethargy was therefore the DLT, and level VII the MTD. Pharmacokinetic data showed no measurable drug interaction; 6/30 patients (20%) had objective responses. This combination is active with manageable toxicity. Recommended doses for further evaluation are irinotecan 350 mg m^–2^and raltitrexed 3.0 mg m^–2^. © 2000 Cancer Research Campaign


*British Journal of Cancer* (2000) **83**(2), 146–152
© 2000 Cancer Research Campaign
doi: 10.1054/ bjoc.2000.1192, available online at http://www.idealibrary.com on IDEAL®

### Phase I study of irinotecan and raltitrexed in patients with advanced gastrointestinal tract adenocarcinoma

**HER Ford[1,2], D Cunningham[1], PJ Ross[1], S Rao[1], GW Aherne[2], TS Benepal[1], T Price[1], A Massey[1], L Vernillet[3] and G Gruia[3]**

[1]Department of Medicine and Gastrointestinal Unit, Royal Marsden NHS Trust, Downs Road, Sutton, Surrey, SM2 5PT, UK; [2]CRC Centre for Cancer Therapeutics, Institute of Cancer Research, 15 Cotswold Road, Sutton, Surrey SM2 5NG, UK; [3]Rhône-Poulenc Récherche et Developement, Antony Cedex, France

**Summary** To determine the dose-limiting toxicities (DLT) and maximum tolerated dose (MTD) of irinotecan and raltitrexed given as sequential short infusions every 3 weeks, 33 patients with pretreated gastrointestinal adenocarcinoma (31 colorectal, 2 oesophagogastric) entered this open label dose-escalation study. For the first five dose levels patients received irinotecan 175–350 mg m$^{-2}$ followed by raltitrexed 2.6 mg m$^{-2}$. Level VI was irinotecan 350 mg m$^{-2}$ plus raltitrexed 3.0 mg m$^{-2}$, level VII was irinotecan 400 mg m$^{-2}$ plus raltitrexed 2.6 mg m$^{-2}$; 261 courses were administered. Only one patient at dose levels I–V experienced DLT. At level VI, 5/12 patients experienced DLT: one had grade 3 diarrhoea and lethargy, one had grade 4 diarrhoea and one had lethargy alone. Two others had lethargy caused by disease progression. There was no first-cycle neutropenia. At level VII, 3/6 patients experienced dose-limiting lethargy, one also had grade 3 diarrhoea. Dose intensity fell from over 90% for both drugs at level VI to 83% for irinotecan and 66% for raltitrexed at level VII. Lethargy was therefore the DLT, and level VII the MTD. Pharmacokinetic data showed no measurable drug interaction; 6/30 patients (20%) had objective responses. This combination is active with manageable toxicity. Recommended doses for further evaluation are irinotecan 350 mg m$^{-2}$ and raltitrexed 3.0 mg m$^{-2}$. © 2000 Cancer Research Campaign

**Keywords:** irinotecan; raltitrexed; drug combination; phase I; pharmacokinetics

Malignant tumours of the gastrointestinal (GI) tract are common, leading to premature death in a high proportion of cases. 5-Fluorouracil (5-FU)-based chemotherapy for advanced disease is superior to best supportive care in colorectal and gastric cancer (Nordic Gastrointestinal Tumour Adjuvant Therapy Group, 1992; Murad et al, 1993). Combination chemotherapy for both upper and lower GI tract cancers is now producing increased response rates with acceptable toxicity (Webb et al, 1997; Ross et al, 1997). New active agents have recently been developed, and attention is focusing on combinations of these in an attempt further to improve response rates and survival. Irinotecan hydrochloride, via its active metabolite SN-38, is an inhibitor of DNA topoisomerase I, an enzyme essential for DNA transcription and commonly overexpressed in colorectal adenocarcinomas (Giovanella et al, 1989). It has in vitro activity against a number of different cell lines (Shimada et al, 1994), and demonstrated activity against a variety of tumours including colorectal and gastric cancers in phase II trials. Irinotecan also displays non cross-resistance to 5-FU (Rougier et al, 1997) and is approved as a single agent for the treatment of 5-FU-refractory colorectal cancer. Dose-limiting toxicities (DLT) are myelosuppression and delayed diarrhoea, which is unpredictable and may be life-threatening (Armand, 1996). Early use of high-dose loperamide can minimize the danger from this effect (Abigerges et al, 1994), and it appears that neutropaenia is the true DLT. Raltitrexed is a specific thymidylate synthase inhibitor (Jackman et al, 1991). It has equivalent response rates to 5-FU plus leucovorin (LV) in advanced colorectal cancer, with reduced antiproliferative toxicity (Cunningham et al, 1996). DLT are gastrointestinal (diarrhoea) and haematological (neutropenia and thrombocytopenia), as well as lethargy (Clarke et al, 1996; Grem et al, 1999). In vitro studies with irinotecan and raltitrexed demonstrate highly sequence-specific synergy (Aschele et al, 1998). The primary objectives of this study were to determine the maximum tolerated doses (MTD) of the two drugs given as short infusions every 3 weeks and to determine the toxic effects and DLT of the combination. Secondary objectives were to measure the pharmacokinetics of irinotecan, SN-38 and raltitrexed, to assess the anti-tumour effect of the combination and to recommend a safe dose for phase II evaluation.

*Received 11 November 1999*
*Revised 15 February 2000*
*Accepted 24 February 2000*

*Correspondence to:* D Cunningham

### METHODS

### Definitions

DLT was defined as any grade 3 or 4 non-haematological toxicity (except alopecia) following cycle 1; grade 4 neutropenia or grade 3–4 neutropenia associated with fever (> 38°C) or sepsis; grade 4 thrombocytopenia or any grade of thrombocytopenia associated with haemorrhage. MTD was defined as the dose level at which 50% of patients experienced the same DLT.

### Eligibility

Eligibility criteria were as follows: histologically confirmed advanced GI tract adenocarcinoma; life expectancy ≥ 12 weeks;

WHO performance status (PS) ≤ 2; age 18–70 years; satisfactory renal and hepatic function; written informed consent.

Exclusion criteria were: previous treatment with topoisomerase I inhibitor; chronic enteropathy; symptomatic cerebral metastasis or leptomeningeal carcinomatosis; unresolved bowel obstruction; pregnancy; previous malignant disease (except adequately treated carcinoma in situ of the cervix uteri or basal cell carcinoma of the skin).

### Pre-treatment evaluation

Prior to entry, patients were evaluated with a physical examination. An ECG, chest X-ray and CT scan of thorax abdomen and pelvis were carried out on all patients. Other radiological investigations were carried out if indicated. A full blood count, renal function tests, liver function tests and prothrombin time were also performed.

### Treatment schedule

Irinotecan was administered as a 30-min infusion on day 1 followed after 30 min by a 15-min infusion of raltitrexed. This cycle was repeated at 21-day intervals. The dose escalation schedule is shown in Table 1. A minimum of three patients were recruited to each dose level, and escalation to the next level was carried out when all three had been observed for a minimum of 2 weeks. The initial design was for the level below MTD to be expanded to six patients. Due to the unusual pattern of toxicities seen, however, the MTD dose level was expanded to six patients, and 12 patients were treated at the level below MTD. Patients received six cycles of treatment subject to favourable response. Further cycles could be administered at the investigators' discretion if there was evidence of continuing clinical benefit.

### Pharmacokinetic measurements

Blood was taken for pharmacokinetic studies after the first cycle of treatment at the following sampling times: before irinotecan administration and then 15, 25, 35, 40 and 45 min; 1, 1.5, 1.75, 2, 2.5, 4.5, 7.5, 9, 13.5, 25.5, 49.5, 73.5, 145.5, 313.5 and 481.5 h after the start of the irinotecan infusion. Irinotecan and SN38 were measured by reversed-phase HPLC using a precipitation step by an acetonitrile-methanol mixture (50/50 v/v) containing the internal standard (camptothecin), and a fluorescence detection at excitation and emission wavelengths of 355 nm and 515 nm respectively for irinotecan, and 388 nm and 540 nm for SN-38. Raltitrexed concentrations were measured by radioimmunoassay (Aherne et al, 1998). Pharmacokinetic analysis was performed using WinNonlin software. The calculation of kinetic parameters was performed using a 2- or 3-compartment open model for irinotecan, and a non-compartmental analysis for its metabolite SN-38 and raltitrexed. The following parameters were evaluated: maximum plasma concentration ($C_{max}$); area under the plasma concentration-time curve (AUC); total body clearance (Cl); volume of distribution at steady state ($V_{ss}$) and terminal half-life ($t_{1/2}$).

**Table 1** Dose escalation scheme

| Dose level | Irinotecan dose (mg m$^{-2}$) | Raltitrexed dose (mg m$^{-2}$) |
|---|---|---|
| I | 175 | 2.6 |
| II | 200 | 2.6 |
| III | 250 | 2.6 |
| IV | 300 | 2.6 |
| V | 350 | 2.6 |
| VI | 350 | 3.0 |
| VII | 400 | 2.6 |

### Evaluation and management of toxicity

No prophylactic antidiarrhoeal treatment was given. Patients who suffered from the cholinergic syndrome (an acute toxicity of irinotecan, consisting of some or all of diarrhoea, hypersalivation, lachrymosis, visual disturbance, diaphoresis and abdominal cramps) were treated with atropine 250 µg by subcutaneous injection, and pretreated with this for each subsequent course. Patients were seen weekly while on treatment. Toxicities were graded according to the NCI common toxicity criteria (NCI-CTC). Delayed diarrhoea was treated with high-dose loperamide: patients were instructed to take 2 mg of loperamide at the onset of diarrhoea, and to take a further 2 mg every 2 h for at least 12 h after the last loose stool. Any patient experiencing concomitant vomiting was hospitalized to prevent dehydration. If diarrhoea persisted for more than 24 h, patients were treated with ciprofloxacin 250 mg b.i.d. In the case of diarrhoea persisting for more than 48 h the patient would be admitted to hospital for parenteral support. All patients with febrile neutropaenia were admitted to hospital and treated with intravenous antibiotics. Doses of both irinotecan and raltitrexed were reduced by 20% if any of the following occurred: grade 4 neutropenia; grade 3 neutropenia with fever; grade 3 or 4 diarrhoea. If day 22 absolute neutrophil count was less than $1.5 \times 10^9$ l$^{-1}$ or platelet count less than $100 \times 10^9$ l$^{-1}$, treatment was delayed up to a maximum of 2 weeks. In the case of myelosuppression lasting more than 5 weeks from the date of treatment, the patient was withrawn from the study. All patients were treated under the auspices of the GI unit at the Royal Marsden Hospital, and the protocol was approved by the hospital's committee for clinical research and research ethics committee.

### Evaluation of response

Responses were evaluated according to standard WHO criteria. Follow up CT scan evaluation was carried out after alternate treatment cycles. Clinical evaluation and assessment of symptoms was carried out prior to each treatment cycle.

### Statistical methods

The results presented consist largely of a descriptive analysis. In addition, Kaplan-Meier curves (Kaplan and Meier, 1958) were generated for overall and progression-free survival, and median time to disease progression (TTP) and overall survival (OS) calculated.

### RESULTS

Between September 1996 and April 1998, 34 patients entered the study (Table 2). One registered patient developed a fever prior to

*© 2000 Cancer Research Campaign*

**Table 2** Patient characteristics

| Characteristic | Number |
|---|---|
| Sex | |
| Male | 24 |
| Female | 9 |
| Age (years) | |
| Median | 56 |
| Range | 38–71 |
| PS (WHO) | |
| 0 | 11 |
| 1 | 19 |
| 2 | 3 |
| Primary site | |
| Colon | 14 |
| Rectum | 17 |
| Oesophagus | 1 |
| Stomach | 1 |
| Prior chemotherapy lines (including adjuvant therapy) | |
| 1 | 24 |
| 2 | 9 |

**Table 3** Patients and courses per dose level

| Dose level | No. of patients | No. of courses (range) |
|---|---|---|
| I | 3 | 43 (3–33) |
| II | 3 | 41 (12–16) |
| III | 3 | 33 (9–12) |
| IV | 3 | 49 (2–30) |
| V | 3 | 13 (2–7) |
| VI | 12 | 45 (1–9) |
| VII | 6 | 37 (2–15) |
| TOTAL | 33 | 261 (1–33) |

starting treatment. By the time his fever had settled, his bilirubin had risen to unacceptably high levels and he was excluded from the study and subsequent analysis. All the other patients were evaluable for toxicity. All had previously been treated with 5-FU-based chemotherapy (5/33 had received adjuvant treatment 7–27 months before relapse and 28/33 had received prior chemotherapy for advanced disease). Patients had received a median of 1 line of prior treatment: 27/33 (82%) had received an infusional 5-FU regimen, while 6/33 had been treated with bolus 5-FU/LV. Of the evaluable patients, 11/25 (44%) had previously had objective responses to 5-FU. 261 cycles were given (median five per patient, range 1–33) all of which were assessable for toxicity (Table 3).

### Toxicity

There were no treatment-related deaths. Toxicities are summarized in Tables 4 and 5. Grade 4 neutropenia was seen in six patients, at dose levels I (two patients), IV, VI (two patients) and VII. It was never seen following the first cycle of treatment and was therefore not dose-limiting. Duration of neutropaenia ranged from 3–8 days. In four cases it was associated with fever requiring hospital admission: two patients developed Hickman line-related septicaemia which resolved on removal of the line and antibiotic therapy; one patient developed *Klebsiella pneumoniae* septicaemia and responded to standard antibiotic treatment; the other patient was admitted with fever, lethargy and grade 2 diarrhoea following cycle 3. No pathogens were isolated from blood, urine or faeces and he made an uneventful recovery following administration of intravenous antibiotics. In one case, grade 4 neutropenia was accompanied by grade 2 diarrhoea and stomatitis in the absence of fever. The patient was admitted to hospital, treated with supportive therapy and intravenous antibiotics, and recovered rapidly. One other patient was found to have asymptomatic grade 4 neutropaenia on a routine blood count. Of these patients, two received no further treatment and the other four had no further neutropaenia episodes following dose reduction. Grade 3 thrombocytopenia was seen after two of 261 courses (one at dose level I, one at dose level VI), grade 3 anaemia occurred after 1 of 261 cycles. There were no incidences of grade 4 anaemia or thrombocytopenia.

Mild (grade 1–2) diarrhoea occurred after 45 of 261 courses (15%). Grade 3 or 4 diarrhoea was only seen in four patients, all at dose levels VI and VII. Even at these dose levels, it was only seen after 3 of 45 (7%) and 1 of 33 (3%) treatment cycles respectively. In one patient it was associated with neutropenia (grade 2) and fever. Lethargy was graded as mild, moderate or severe (grades 1–3). Increasingly severe lethargy was seen at higher dose levels. Nine of 12 patients at dose level VI and 5/6 patients at dose level VII experienced severe (grade 3) lethargy. At dose level VII, the onset of symptoms was earlier (with 3/6 patients experiencing lethargy following their first treatment cycle, compared with 4/12 at level VI and only one patient at lower dose levels) and more prolonged. Grade 3 lethargy was therefore defined as a DLT. The number of patients experiencing severe lethargy was of concern, and dose levels VI and VII were therefore expanded to 12 and 6 patients respectively, with the aim of further characterizing this toxicity. In six of the nine patients experiencing grade 3 lethargy at dose level VI, the symptom was due to progressive disease. Despite the absence of dose reduction, four of the nine patients' lethargy improved on treatment and subsequently returned at the time of disease progression, suggesting a disease-related phenomenon. Two further patients had disease which progressed rapidly after two cycles of treatment, and it was felt that their lethargy was principally attributable to disease. In only two patients at this dose level was lethargy clearly drug-related. In one further patient, lethargy was associated with disease progression, but improved on cessation of treatment, and this patient was regarded as having drug-related lethargy. At dose level VII, five out of six patients experienced grade 3 lethargy, and in four the symptom was clearly attributable to toxicity, with significant improvement on cessation or dose reduction.

Mild nausea was common (27/33 patients), but easily controlled with standard anti-emetic medication. Elevation of hepatic transaminases occurred in most patients but tended to improve in subsequent courses and was not dose-limiting. In one patient transaminitis failed to resolve, and he was withdrawn from the study. CT scan after a further two cycles of irinotecan showed progressive disease in the liver, which was felt to be responsible. One patient developed lower limb cellulitis secondary to raltitrexed, and was treated with irinotecan alone for 6 cycles. This patient subsequently received a further four cycles of the irinotecan/raltitrexed combination without complication. Two patients developed grade 1 rises in serum creatinine, necessitating dose delay. One of these had a hydronephrosis secondary to peritoneal metastases. Following ureteric stenting, his creatinine

*© 2000 Cancer Research Campaign*

**Table 4** NCI-CTC grade 1–2 toxicity by dose level (all cycles)

| Dose level | Lethargy (mild–moderate) | NCI-CTC toxicity grade 1–2 | | | |
|---|---|---|---|---|---|
| | | Neutropenia | Diarrhoea | Nausea | Transaminitis |
| I | 3/3 | 0/3 | 3/3 | 2/3 | 1/3 |
| II | 3/3 | 1/3 | 0/3 | 3/3 | 2/3 |
| III | 2/3 | 0/3 | 3/3 | 3/3 | 1/3 |
| IV | 1/3 | 0/3 | 3/3 | 3/3 | 2/3 |
| V | 3/3 | 1/3 | 2/3 | 2/3 | 0/3 |
| VI | 2/12 | 3/12 | 6/12 | 11/12 | 6/12 |
| VII | 0/6 | 2/6 | 4/6 | 3/6 | 3/6 |

**Table 5** NCI-CTC grade 3–4 toxicity by dose level (all cycles)

| Dose level | Lethargy (severe) | NCI-CTC toxicity grade 3–4 | | | |
|---|---|---|---|---|---|
| | | Neutropenia | Diarrhoea | Nausea | Transaminitis |
| I | 0/3 | 2/3 | 0/3 | 0/3 | 1/3 |
| II | 0/3 | 0/3 | 0/3 | 0/3 | 1/3 |
| III | 1/3 | 1/3 | 0/3 | 0/3 | 1/3 |
| IV | 2/3 | 3/3 | 0/3 | 0/3 | 0/3 |
| V | 0/3 | 0/3 | 0/3 | 0/3 | 2/3 |
| VI | 9/12 | 6/12 | 3/12 | 1/12 | 5/12 |
| VII | 5/6 | 2/6 | 1/6 | 1/6 | 3/6 |

**Table 6** First-cycle toxicity

| Dose level | Lethargy G3 | Neutropenia G4 | Diarrhoea G3–4 | Total no. of patients experiencing DLT |
|---|---|---|---|---|
| I | 0/3 | 0/3 | 0/3 | 0 |
| II | 0/3 | 0/3 | 0/3 | 0 |
| III | 0/3 | 0/3 | 0/3 | 0 |
| IV | 1/3 | 0/3 | 0/3 | 1 |
| V | 0/3 | 0/3 | 0/3 | 0 |
| VI | 4/12[a] | 0/12 | 2/12 | 5[b] |
| VII | 3/6 | 0/6 | 1/6 | 3 |

[a]Drug-related toxicity in 2/4 patients; [b]Drug-related toxicity in 3/12 patients improved sufficiently to allow further chemotherapy. The other patient was found to have a creatinine clearance of 67 ml $min^{-1}$, and her serum creatinine when measured 7 days later was normal. She therefore continued treatment as scheduled. A summary of first-cycle toxicities by dose level is shown in Table 6. It was concluded that the DLT for the combination of irinotecan and raltitrexed is lethargy, and that the MTD is irinotecan 400 mg $m^{-2}$ and raltitrexed 2.6 mg $m^{-2}$ 3-weekly. The recommended doses for phase II evaluation were set at irinotecan 350 mg $m^{-2}$ and raltitrexed 3.0 mg $m^{-2}$.

### Dose intensity

Calculations were made from the first six treatment cycles. Dose delays were seen after 24/143 cycles (17%). Reasons for delay were: raised hepatic transaminases (8/24); neutropenia (5/24); lethargy (4/24); infection without neutropenia (2/24); elevated serum creatinine (2/24); patient request (2/24) and subacute bowel obstruction (1/24). Median dose delay was 7 days at dose levels I-VI and 12 days at level VII. Six patients required dose reduction. Dose intensity (DI) for each drug was calculated as a percentage of intended. DI approximated 100% at levels I-VI. At level VII, raltitrexed DI fell to 66% and irinotecan DI was only 83%, providing further evidence for this being the MTD.

### Irinotecan pharmacokinetics

Pharmacokinetic data for irinotecan was obtained in 26 patients (Table 7). The total plasma clearance of irinotecan was relatively stable over the seven investigated dose levels with an overall mean value of 12.4 l $h^{-1}$ $m^{-2}$. A slightly higher interpatient variability was observed for the other parameters. For SN-38, maximal concentrations were mainly observed within the 60 min following the end of i.v. infusion, and the apparent terminal half-life was stable over the administered dose range with a mean value of 11.1 h.

© 2000 Cancer Research Campaign

**Table 7** Pharmacokinetic parameters for irinotecan and SN-38

| Dose level | No. of patients | Irinotecan | | | | | SN-38 | | SN-38/irinotecan AUC ratio (%) |
|---|---|---|---|---|---|---|---|---|---|
| | | $C_{max}$[a] (μg ml$^{-1}$ | AUC (μg h ml$^{-1}$) | Cl (l h$^{-1}$ m$^{-2}$) | $V_{ss}$ (l m$^{-2}$) | $t_{\frac{1}{2}}$ terminal (h) | $C_{max}$[b] (μg ml$^{-1}$) | AUC (μg h ml$^{-1}$) | |
| I | 3 | 3.53 ± 0.07 | 13.1 ± 1.6 | 11.7 ± 1.6 | 63 ± 9 | 5.5 ± 2.2 | 0.048 ± 0008 | 0.27 ± 0.12 | 2.1 ± 0.8 |
| II | 3 | 4.70 ± 0.56 | 16.0 ± 5.4 | 11.6 ± 3.5 | 9.8 ± 1.2 | 9.8 ± 2.2 | 0.074 ± 0.048 | 0.52 ± 0.25 | 3.2 ± 0.6 |
| III | 3 | 5.92 ± 1.15 | 14.6 ± 1.8 | 15.0 ± 2.0 | 11.7 ± 7.2 | 11.7 ± 7.2 | 0.060 ± 0.014 | 0.33 ± 0.03 | 2.3 ± 0.1 |
| IV | 3 | 5.98 ± 4.22 | 22.6 ± 6.2 | 12.6 ± 3.4 | 11.4 ± 5.0 | 11.4 ± 5.0 | 0.071 ± 0.017 | 0.44 ± 0.10 | 2.1 ± 0.8 |
| V | 3 | 4.89 ± 1.03 | 26.6 ± 11.4 | 12.5 ± 4.6 | 7.2 ± 0.9 | 7.2 ± 0.9 | 0.057 ± 0.015 | 0.78 ± 0.35 | 2.9 ± 0.3 |
| VI | 6 | 5.43 ± 1.00 | 29.0 ± 12.2 | 11.3 ± 3.1 | 9.6 ± 3.9 | 9.6 ± 3.9 | 0.100 ± 0.016 | 0.58 ± 0.23 | 2.1 ± 0.8 |
| VII | 5 | 5.41 ± 2.60 | 27.0 ± 7.4 | 12.9 ± 2.7 | 10.7 ± 6.6 | 10.7 ± 6.6 | 0.117 ± 0.116 | 0.76 ± 0.39 | 2.7 ± 0.9 |

[a]estimated $C_{max}$ at the end of i.v. infusion; [b]observed $C_{max}$.

**Table 8** Pharmacokinetic parameters for raltitrexed

| Dose level | No. of patients | $C_{max}$ (ng ml$^{-1}$) | AUC (ng h ml$^{-1}$) | Cl (l h$^{-1}$) | $V_{ss}$ (l) | $t_{\frac{1}{2}}$ terminal (h) |
|---|---|---|---|---|---|---|
| I | 3 | 561 ± 78 | 1711 ± 607 | 3.01 ± 0.77 | 40.7 ± 14.8 | 222 ± 102 |
| II | 3 | 420 ± 72 | 1440 ± 697 | 4.70 ± 1.9 | 27.3 ± 6.1 | 190 ± 107 |
| III | 3 | 291 ± 32 | 1464 ± 305 | 3.36 ± 0.58 | 60.2 ± 31.4 | 345 ± 62 |
| IV | 3 | 676 ± 185 | 1814 ± 381 | 2.83 ± 0.86 | 32.3 ± 15.5 | 348 ± 171 |
| V | 3 | 736 ± 161 | 2520 ± 1586 | 2.50 ± 1.4 | 30.2 ± 6.7 | 403 ± 99 |
| VI | 6 | 772 ± 204 | 2480 ± 698 | 2.41 ± 0.90 | 25.8 ± 9.4 | 296 ± 16 |
| VII | 6 | 574 ± 143 | 1503 ± 177 | 3.34 ± 0.86 | 43.8 ± 9.6 | 261 ± 73 |

### Raltitrexed pharmacokinetics

Pharmacokinetic data was obtained from 27 patients (Table 8). There were minor variations in individual parameters with increasing doses of irinotecan, however Cl was stable at 3.1 l h$^{-1}$ over all dose ranges, as was the $t_{1/2}$ at 291 h.

### Survival

Twenty-six patients have died and two are lost to follow-up. With a median follow-up of 14.8 months, median survival is 9.3 months. Median progression-free survival is 4.8 months.

### Response

Thirty patients had measurable disease. Of these there were six partial responses, giving an objective response rate of 20%. Median response duration was 6.9 months. Thirteen patients (43%) had stable disease confirmed by CT scans 6 weeks apart. The median time to disease progression (TTP) in these patients is 7.7 months. Eleven patients (37%) progressed, including both of the patients with oesophagogastric cancer. Responses were seen at dose levels I, II, IV, VI (two responses) and VII.

### DISCUSSION

Combination therapy is likely to represent the future standard of care for advanced colorectal cancer. Phase I studies of irinotecan in combination with 5-FU have demonstrated that effective doses of the two drugs may be given together despite the potential for overlapping toxicities (Saltz et al, 1996). Recently completed phase III studies have shown that this combination provides improved response rates when compared to 5-FU alone (Saltz et al, 1999; Douillard et al, 1999). This study aimed to assess the potential of the similar combination of irinotecan and raltitrexed. The in vitro data showing synergy when cells were exposed to SN-38 before raltitrexed provided the rationale for giving the drugs in this sequence. One preclinical study suggested that this synergy was further potentiated by a 24 h interval between administration of the drugs (Aschele et al, 1998), however same day administration was chosen for this trial for patient convenience. For irinotecan, the pharmacokinetic results were consistent with previous monotherapy data (Abigerges et al, 1995). Although total body clearance was slightly lower at the highest dose investigated of 400 mg m$^{-2}$ (12.9 vs 14.8 l h$^{-1}$ m$^{-2}$), other parameters were comparable (Vss 146 vs 150 l m$^{-2}$ and terminal $t_{1/2}$ 10.7 vs 12.0 h). SN-38 $t_{1/2}$ was also similar to that determined in 168 phase I cancer patients treated with irinotecan alone (11.1 vs 10.6 h). SN-38 Cmax and AUC values were in the same range as with irinotecan monotherapy (e.g. at the highest dose tested 0.117 vs 0.094 μg ml$^{-1}$ and 0.76 vs 0.67 μg h$^{-1}$ ml$^{-1}$ respectively) (Chabot et al, 1995). Despite the administration of raltitrexed, SN-38/irinotecan AUC ratio values were roughly stable over the tested dose range, and were close to those observed in monotherapy (overall mean value of 2.4 vs 3.1%) (Abigerges et al, 1995). There is less published data available for raltitrexed. However in this trial, despite patient-to-patient variability, results were similar to those obtained with single-agent treatment. For instance at 3.0 mg m$^{-2}$, mean values for raltitrexed Cmax and AUC were 772 vs 737 ng ml$^{-1}$ and 2480 vs 2342 ng h$^{-1}$ ml$^{-1}$ respectively, when compared to data obtained from the administration of $^{14}$C labelled raltitrexed (Beale et al, 1998). At this dose level, values for raltitrexed clearance (2.41 vs

 © 2000 Cancer Research Campaign

2.48 l $h^{-1}$ and $t_{1/2}$ (296 vs 257 h) were also consistent with data from this study. These data suggest that the concomitant administration of raltitrexed and irinotecan according to this schedule does not modify the behaviour of either drug.

Since this study was completed, work has been published showing that the AUC for raltitrexed may be approximately doubled in patients with measured creatinine clearance less than 65 ml $min^{-1}$ (Judson et al, 1998). Entry into this study was based on normal serum creatinine (or normal measured creatinine clearance if serum creatinine was elevated). In the light of this information, however, patients' calculated creatinine clearance (using the Cockroft and Gault formula) was retrospectively examined. Only one patient had a pre-treatment creatinine clearance less than 65 ml $min^{-1}$ (44 ml $min^{-1}$), and this patient did not experience any toxicity. One patient with grade 3 diarrhoea had a fall in creatinine clearance from 68 to 55 ml $min^{-1}$ following his first cycle of treatment, and did develop asymptomatic and short-lived grade 4 neutropenia following cycle 3. Whether or not these events are related is uncertain, and all other patients experiencing grade 3 or 4 toxicity had normal calculated creatinine clearances before and after treatment. Nevertheless, it is now recommended that patients receiving raltitrexed have creatinine clearance calculated prior to each course of treatment and appropriate dose modifications adopted in the case of values less than 65 ml $min^{-1}$, and this recommendation should also be applied to the use of the drug in combination. Similarly, the clearance of irinotecan is predominantly liver-dependent (Raymond et al, 1999), and there is a theoretical possibility that, although it seldom if ever causes elevation of bilirubin, the transaminitis induced by raltitrexed might affect irinotecan pharmacokinetics. There was no association in this study between transaminitis and other toxicities. In addition, toxicity overall was no greater than might have been expected from single-agent irinotecan, despite the high incidence of transaminitis seen (85% of all patients).

Although pharmacokinetics were not tested for the second and subsequent cycles, and it is impossible therefore to know whether or not pharmacokinetics are affected, there is no evidence that raltitrexed-induced transaminitis increases susceptibility to toxicity from this combination.

In this study it is notable that diarrhoea and myelosuppression, which are the DLT for both drugs as single agents, were not a major feature of the combination. The DLT of this combination is lethargy. This may occur at any dose, but at the higher dose levels, especially level VII, was often much more severe and prolonged. When the protocol was drawn up there was no CTC scale for lethargy, although the severe lethargy seen at these levels corresponds to grade 4 toxicity in the latest CTC revision (CTC version 2.0, National Cancer Institute, Bethesda, January 1998). Unfortunately, it is often difficult to differentiate lethargy caused by disease progression from that caused by the drugs themselves, and this inevitably introduces an element of subjectivity into the interpretation of the results. To reduce this as far as possible grade 3 (severe) lethargy was therefore defined as a DLT, regardless of cause. When interpreting the palliative benefits of the regimen, however, it is important to note that of nine cases of grade 3 lethargy seen at dose level VI, six were associated with simultaneous disease progression. Only two patients at this dose level had grade 3 lethargy which was clearly attributable to the study regimen, and indeed in four of the other cases (none of whom had a dose reduction) lethargy improved in subsequent cycles, only to re-emerge at the time of disease progression. By contrast, at dose level VII only one patient did not suffer from severe lethargy, and of the five who did, only one had associated disease progression. The remaining four patients' lethargy improved on cessation of treatment or following dose reduction. It was to evaluate this toxicity further that dose levels VI and VII were expanded to 12 and 6 patients respectively, and we are satisfied that although severe lethargy may be seen at dose level VI, it is only dose-limiting at level VII. In view of the phase I data showing increased lethargy with raltitrexed at doses above 3.0 mg $m^{-2}$ (Clarke et al, 1996) it was decided not to further escalate the raltitrexed dose. Further support for level VII as the MTD is provided by the dose intensity figures, with a major fall in DI at this dose level. The recommended dose for phase II evaluation was therefore set at level VI (irinotecan 350 mg $m^{-2}$ and raltitrexed 3.0 mg $m^{-2}$) despite the absence of conventional DLT.

The definition of MTD chosen for this study was the dose level at which 50% of patients experienced the same DLT. This apparently aggressive definition was chosen because experience in phase I studies of raltitrexed had shown that a variety of sporadic grade 3 and 4 toxicities could occur at lower dose levels (Clarke et al, 1996) and we did not wish to terminate the trial prematurely following the occurrence of three different non dose-related toxicities. In retrospect, a more conventional definition may have been appropriate for the study. Nevertheless, the MTD would in fact have been the same using either definition, and there is therefore no suggestion that the choice of definition affected the validity of the results.

There was evidence of anti-tumour activity, with an ORR of 20% in predominantly 5-FU-refractory patients, and a significant proportion of patients (43%) had disease stabilization, often for a prolonged period. Another issue raised by the study is the optimum duration of treatment. Eleven patients on this study (33%) received more than eight cycles of treatment. Indeed, one patient received 33 cycles without a partial response prior to disease progression. Interestingly, he was being treated at low doses of the combination (dose level I). Preclinical models have suggested that irinotecan has an anti-angiogenic effect (O'Leary et al, 1999), and this is one possible explanation. At the moment there are no guidelines as to the optimum duration of treatment with irinotecan, although clinical studies are addressing this question. Related to this is the relevance of disease stabilization as a therapeutic goal. Over 40% of patients in this trial achieved stable disease, and although this may merely indicate a subset of patients with indolent tumours, the median TTP of 7.7 months in this group compared to 6.9 months in responding patients does suggest significant clinical benefit.

In conclusion, the combination of irinotecan and raltitrexed is active and well tolerated, and the 3-weekly schedule is convenient for patients. This combination merits further investigation, and a phase II study in colorectal cancer has been initiated at the doses of irinotecan 350 mg $m^{-2}$ and raltitrexed 3.0 mg $m^{-2}$ every 3 weeks.

### ACKNOWLEDGEMENTS

We would like to thank A Lecuit, O Pasquier, N Taslaud, JC Vergniol and T Crompton for their technical assistance.

### REFERENCES

Abigerges D, Armand JP, Chabot GG, Da Costa L, Fadel E, Cote C, Herait P and Gandia D (1994) Irinotecan (CPT-11) high dose escalation using intensive high-dose loperamide to control diarrhoea. *J Natl Cancer Inst* **86**: 446–449

© 2000 Cancer Research Campaign 

Abigerges D, Chabot GG, Armand JP, Herait P, Gouyette A and Gandia D (1995) Phase I and pharmacologic studies of the camptothecin analog irinotecan administered every 3 weeks in cancer patients. *J Clin Oncol* **13**: 210–221

Aherne GW, Ward E, Lawrence N, Dobinson D, Clarke SJ, Musgrove H, Sutcliffe F, Stephens T and Jackman AL (1998) Comparison of plasma and tissue levels of ZD1694 (Tomudex), a highly polyglutamable quinazoline thymidylate synthase inhibitor in preclinical models. *Br J Cancer* **77**: 221–226

Armand JP (1996) CPT-11: clinical experience in phase I studies. *Semin Oncol* **23** (suppl. 3): 27–33

Aschele C, Baldo C, Sobrero A, Debernardis D, Bornmann WG and Bertino JR (1998) Schedule dependent synergism between raltitrexed and irinotecan in human colon cancer cells *in vitro*. *Clinical Cancer Research* **4**: 1323–1330

Beale P, Judson I, Hanwell J, Berry C, Aherne W, Hickish T, Martin P and Walker M (1998) Metabolism, excretion and pharmacokinetics of a single dose of [14C]-raltitrexed in cancer patients. *Canc Chemother Pharmacol* **42**: 71–76

Chabot GG, Abigerges D, Cartimel G, Culine S, de Forni M, Extra JM, Mahjoubi M, Herait P, Armand JP and Bugat R (1995) Population pharmacokinetics and pharmacodynamics of irinotecan (CPT-11) and active metabolite SN-38 during phase I trials. *Ann Oncol* **6**: 141–151

Clarke SJ, Hanwel J, De Boer M, Planting A, Verweij J, Walker M, Smith R, Jackman AL, Hughes LR, Harrap KR, Kennealy GT and Judson IR (1996) Phase I trial of ZD1694, a new folate-based thymidylate synthase inhibitor, in patients with solid tumours. *J Clin Oncol* **14**: 1495–1503

Cunningham D, Zalcberg JR, Rath U, Van Cutsem E, Svensson C, Seitz JF, Harper P, Kerr D and Perez Manga G (1996) Final results of a randomised trial comparing 'Tomudex' (raltitrexed) with 5-fluorouracil plus leucovorin in advanced colorectal cancer. *Ann Oncol* **7**: 961–965

Douillard JY, Cunningham D, Roth AD, Navarro M, James RD, Karasek P, Jandik P, Iveson T, Carmichael J, Alakl M, Gruia G, Awad L and Rougier P. A randomised phase III trial comparing irinotecan (IRI) + 5FU/folinic acid (FA) to the same schedule of 5FU/FA in patients with metastatic colorectal cancer (MCRC) as frontline therapy. *Lancet* **355**:1041–1047

Giovanella BC, Stehlin JS, Wall ME, Wani MC, Nicholas AW, Liu LF, Silber R and Potemsil M (1989) DNA topoisomerase l-targeted chemotherapy of human colon cancer in xenografts. *Science* **246**: 1046–1048

Grem JL, Sorensen JM, Cullen E, Takimoto CH, Steinberg SM, Chen AP, Hamilton JM, Arbuck SG, McAtee N, Lawrence D, Goldspiel B, Johnston PG and Allegra CJ (1999) A phase I study of raltitrexed, an antifolate thymidylate synthase inhibitor, in adult patients with advanced solid tumors. *Clinical Cancer Research* **5**: 2381–2391

Jackman AL, Taylor GA, Gibson W, Kimbell R, Brown M, Calvert AH, Judson IR and Hughes LR (1991) ICI DI694, a quinazoline antifolate thymidylate synthase inhibitor that is a potent inhibitor of L1210 tumor cell growth *in vitro* and *in vivo*: a new agent for clinical study. *Cancer Res* **51**: 5579–5586

Judson I, Maughan TS, Beale P, Primrose J, Hoskin P, Hanwell J, Berry C, Walker M and Sutcliffe F (1998) Effects of impaired renal function on the pharmacokinetics of raltitrexed (Tomudex, ZD1694). *Br J Cancer* **78(9)**: 1188–1193

Kaplan EL and Meier P (1958) Nonparametric estimation from incomplete observations. *J Am Stat Assoc* **53**: 457–481

Murad AM, Santiago FF, Petroianu A, Petroianu A, Rocha PRS, Rodrigues MAG and Rausch M (1993) Modified therapy with 5-fluorouracil, doxorubicin and methotrexate in advanced gastric cancer. *Cancer* **72**: 37–41

Nordic Gastrointestinal Tumor Adjuvant Therapy Group (1992) Expectancy or primary chemotherapy in patients with advanced asymptomatic colon cancer: a randomized trial. *J Clin Oncol* **10**: 904–911

O'Leary JJ, Shapiro RL, Ren CJ, Chuang N, Cohen HW and Potemsil M (1999) Antiangiogenic effects of camptothecin analogs 9-amino-10(S)-camptothecin, topotecan and CPT-11 studied in the mouse cornea model. *Clinical Cancer Research* **5(1)**: 181–187

Raymond E, Vernillet L, Boige V, Hua A, Ducreux M, Faivre S, Jacques C, Gatineau D, Mignard D, Vergniol JC, Rixe O and Armand JP (1999) Phase I and Pharmacokinetic (PK) Study of Irinotecan (CPT-11) in Cancer Patients (pts) with Hepatic Dysfunction. *Proc Am Soc Clin Oncol* **18**: 165a (abstract 634)

Ross PJ, Norman A, Cunningham D, Webb A, Iveson T, Padhani A, Prendiville J, Watson M, Massey A, Popescu R and Oates J (1997) A prospective randomised trial of protracted venous infusion 5-fluorouracil with or without mitomycin C in advanced colorectal cancer. *Ann Oncol* **8**: 995–1001

Rougier P, Bugat R, Douillard JY, Culine S, Suc E, Brunet P, Becouarn Y, Ychou M, Marty M and Extra JM (1997) Phase II study of irinotecan in the treatment of advanced colorectal cancer in chemotherapynaïve patients and patients pretreated with fluorouracil based chemotherapy. *J Clin Oncol* **15**: 251–260

Saltz LB, Kanowitz J, Kemeny NE, Schaak L, Spriggs D, Staton BA, Berkery R, Steger C, Eng M and Dietz A (1996) Phase I clinical and pharmacokinetic study of irinotecan, fluorouracil and leucovorin in patients wit advanced solid tumors. *J Clin Oncol* **14**: 2959–2967

Saltz LB, Locker PK, Pirotta N, Elfring GL and Miller LL (1999) Weekly irinotecan (CPT-11), leucovorin (LV) and fluorouracil (FU) is superior to daily x5 LV/FU in patients with previously untreated metastatic colorectal cancer. *Proc Am Soc Clin Oncol* **18**: 233a (abstract 898)

Shimada Y, Rothenberg M, Hilsenbeck SG, Burris HA, Degen D and Von Hoff D (1994) Activity of CPT-11 (irinotecan hydrochloride), a topoisomerase I inhibitor, against human tumor colony-forming units. *Anticancer Drugs* **5**: 202–206

Webb A, Cunningham D, Howard Scarffe J, Harper P, Norman A, Joffe JK, Hughes M, Mansi J, Findlay M, Hill A, Oates J, Nicolson M, Hickish T, O'Brien M, Iveson T, Watson M, Underhill C, Wardley A and Meehan M (1997) Randomised trial comparing epirubicin, cisplatin, and fluorouracil versus fluorouracil, doxorubicin and methotrexate in advanced esophagogastric cancer. *J Clin Oncol* **15**: 261–267

 © 2000 Cancer Research Campaign
